# A mixed-methods study to investigate feasibility and acceptability of an early warning score for preterm infants in neonatal units in Kenya: results of the NEWS-K study: Neonatal early warning scores in Kenya

**DOI:** 10.1186/s12887-024-04778-z

**Published:** 2024-05-11

**Authors:** Eleanor J Mitchell, Jalemba Aluvaala, Lucy Bradshaw, Jane P Daniels, Caren Emadau, Bernadine Muthumbi, Helen Nabwera, Ednah Ojee, Jacqueline Opira, Phoebe Pallotti, Zahida Qureshi, Mark Sigei, Yuanfei Su, Richard Swinden, Fredrick Were, Shalini Ojha

**Affiliations:** 1 Nottingham Clinical Trials Unit, School of Medicine, University of Nottingham, Nottingham, UK; 2 Department of Paediatrics, University of Nairobi, Nairobi, Kenya; 3 Department of Paediatrics, Pumwani Maternity Hospital, PO Box 42849, Nairobi, 00100 Kenya; 4 Department of Paediatrics, Thika Level 5 Hospital, PO Box 227, Thika, 001000 Kenya; 5 Centre of Excellence for Women and Child Health, Aga Khan University, Nairobi, Kenya; 6 Kenya Paediatric Research Consortium (KEPRECON), Nairobi, Kenya; 7 Maternal Health and Wellbeing Research Group, School of Health Sciences, University of Nottingham, Nottingham, UK; 8 Department of Obstetrics and Gynaecology, University of Nairobi, Nairobi, Kenya; 9 Academic Unit of Population and Lifespan Sciences, School of Medicine, University of Nottingham, Nottingham, UK

**Keywords:** Early warning score, Neonatal, Newborn unit

## Abstract

Preterm birth (< 37 weeks gestation) complications are the leading cause of neonatal mortality. Early-warning scores (EWS) are charts where vital signs (e.g., temperature, heart rate, respiratory rate) are recorded, triggering action. To evaluate whether a neonatal EWS improves clinical outcomes in low-middle income countries, a randomised trial is needed. Determining whether the use of a neonatal EWS is feasible and acceptable in newborn units, is a prerequisite to conducting a trial.

We implemented a neonatal EWS in three newborn units in Kenya. Staff were asked to record infants’ vital signs on the EWS during the study, triggering additional interventions as per existing local guidelines. No other aspects of care were altered. Feasibility criteria were pre-specified. We also interviewed health professionals (*n* = 28) and parents/family members (*n* = 42) to hear their opinions of the EWS.

Data were collected on 465 preterm and/or low birthweight (< 2.5 kg) infants. In addition to qualitative study participants, 45 health professionals in participating hospitals also completed an online survey to share their views on the EWS. 94% of infants had the EWS completed at least once during their newborn unit admission. EWS completion was highest on the day of admission (93%). Completion rates were similar across shifts. 15% of vital signs triggered escalation to a more senior member of staff. Health professionals reported liking the EWS, though recognised the biggest barrier to implementation was poor staffing. Newborn unit infant to staff ratios varied between 10 and 53 staff per 1 infant, depending upon time of shift and staff type.

A randomised trial of neonatal EWS in Kenya is possible and acceptable, though adaptations are required to the form before implementation.

**Supplementary Information:**

The online version contains supplementary material available at 10.1186/s12887-024-04778-z.

## Introduction

Fifteen million babies are born preterm (before 37 completed weeks of gestation) each year globally 1).Preterm birth complications are the leading cause of death in the first month of life 2). In Kenya, a low-middle income country (LMIC) (3), around 200,000 infants are born preterm each year and the neonatal mortality rate (NMR, infants < 28 days) is high at 21/1000 live births (4). The United Nations Sustainable Development Goals (SDGs) target is to reduce the global NMR to 12/1000 live births by 2030 (5). Provision of neonatal care in Kenya is often sub-optimal with challenges including under-staffing and lack of training for staff leading to inadequate routine assessment of vital signs and poor record-keeping(6,7). Our previous single-centre prospective observational study of 294 preterm infants showed fewer than half the infants had a temperature recorded in medical records on the day of birth (8). Throughout the study period, 63% (*n* = 185) of infants had any temperature recorded of which over half were moderately hypothermic (< 36 °C).

An early warning score (EWS) is a paper-based document used to record patient’s vital signs (9). An EWS utilises a traffic-light system, with red, amber and green zones to visually alert health professionals to abnormal vital signs that should trigger action and the need for closer monitoring. EWS could help improve record-keeping and communication between health professionals and parents, and aid early identification of a clinical deterioration, helping to focus limited resources on those who are at highest risk, and potentially, improving outcomes. Although several neonatal EWSs exist (9,10), none are in widespread use in Kenya or other LMICs. We previously showed that neonatal EWSs could be useful in neonatal care in Kenya, though in the previous study we did not implement an intervention (i.e. the EWS). Data was recorded from medical records and retrospectively plotted onto an EWS at the data analysis stage. Use of an EWS was supported by local stakeholders, although they recognised implementation challenges, including limited staff (8,11).

The aim of this mixed-methods study was to test the feasibility and acceptability of implementation of an EWS for preterm infants in several newborn units in Kenya. The study included prospective quantitative observational data collection and qualitative interviews and focus groups. The study was conducted to inform the design of a randomised controlled trial in the future to evaluate the effectiveness of the EWS on improving neonatal outcomes.

This study is reported in accordance with The Strengthening the Reporting of Observational Studies in Epidemiology (STROBE) statement (12).

## Methods

### Quantitative data collection

#### Study design and setting

We conducted a prospective observational study to test the feasibility of an EWS, in three newborn units in public sector hospitals in Kenya: the Kenyatta National Referral Hospital (the national referral hospital, Nairobi City, providing level 3 neonatal care), Thika Level 5 Hospital (a county referral hospital, Kiambu County, providing level 2 neonatal care) and Mama Lucy Kibaki Hospital (a county hospital, Nairobi County, providing level 2 neonatal care). These hospitals also represent different levels of resources and health care in the Kenyan health system (13). Nurse to infant ratios in newborn units in public sector hospitals vary substantially (14). Hospitals that provide level 2 neonatal care in this setting generally have access to bubble continuous positive airway pressure (CPAP) but have no access to continuous cardiorespiratory monitoring, whereas hospitals that provide level 3 neonatal care provide mechanical ventilation and continuous monitoring.

#### Training

Health professionals in participating units and research staff completed a self-directed bespoke online training module, (designed by the Nottingham Clinical Trials Unit (NCTU), using Xerte software (www.xerte.org.uk)) including generic research and study protocol specific content prior to study start. Further training was conducted virtually using Microsoft Teams©. These sessions included example case studies of how and when to complete the EWS and escalate care appropriately. A total of six training sessions were conducted throughout June and July 2021.

#### Participants and data collection

We used a modified version of the Comprehensive Newborn Monitoring Chart (CNMC) (15) which was being introduced by the Kenyan Ministry of Health at the time of study-set-up. The CNMC does not include a traffic-light system. Our adaptation (NEWS-K CNMC form, supplementary material 1) includes the CNMC, with traffic-light system and guide for escalation of care on the reverse. Staff were advised and trained to continue clinical care as per usual practice consistent with local guidelines.

Our definition of a preterm infant was any infant born < 37 weeks’ gestation and/or < 2.5 kg birthweight. Gestational age was assessed as per usual care in the participating hospitals, which is typically by last menstrual period (LMP). To be eligible for participation, the infant had to be an in-patient in a newborn unit at a participating hospital, irrespective of place of birth. Clinical staff were asked to complete the NEWS-K-CNMC form for all eligible infants, every day for the duration of their newborn unit admission. Observations were conducted in accordance with usual care onto a single NEWS-K CNMC form every day. Basic demographic details were collected for all eligible infants, irrespective of completion of the NEWS-K CNMC form. Clinical characteristics and outcome at discharge were collected on a separate form. A daily log of all admitted infants and staff numbers was completed to measure staff resources available, as previous work showed that inadequate staffing could be a major challenge in use of any EWS (8). The study opened on 16th July 2021 and closed to data collection on 17th September 2021. All newborn unit staff were then invited to complete an online survey, which included a mixture of closed and open questions (supplementary material 2) about their opinions of using the EWS.

#### Outcomes

Outcomes measuring feasibility of use of the NEWS-K-CNMC form were (i) proportion of eligible infants who had observations recorded on the NEWS-K-CNMC each day from admission until discharge or death, of all those admitted to the newborn unit, (ii) number and timing (according to staffing shift, e.g. morning, afternoon, night) of observations per day, (iii) reasons for non-completion of the NEWS-K-CNMC form, where relevant iv) proportion of abnormal vital signs that were acted upon in accordance with the EWS guidance (v) outcomes of reviews triggered by the system, vi) infant’s outcome at discharge or death. For clarity, results are presented separately as clinical outcomes, completion outcomes and escalation outcomes. Pre-specified feasibility thresholds were determined prior to data collection (supplementary material S3).

#### Sample size

Based on existing data, approximately 4150 births were estimated at the three units over six weeks, which was our intended data collection period. Of these births, it was expected that around 10% would be eligible and therefore our aim was to collect data on 432 infants in total, enabling estimation of percentages with a margin of error (half-width of 95% confidence interval) of around 5% points. Due to a lower number of eligible infants than anticipated, the data collection period was extended to 9 weeks.

#### Statistical methods and analysis

A pre-specified statistical analysis plan was developed and approved prior to analyses (supplementary material 4). Pooled data are presented. *C*ompletion of the NEWS-K-CNMC form outcomes are summarised descriptively including where information is unknown on escalation of care. Clinical characteristics are summarised as percentages, mean and standard deviation (SD), or median and interquartile range (IQR) or range as appropriate with number of unknown/missing values if applicable. Analyses were conducted in Stata© (version 17) (StataCorp LLC).

### Qualitative data collection

The qualitative study aimed to test the acceptability of an EWS in newborn units in Kenya and is being reported in full separately. Here we briefly report the data relating to the barriers and facilitators of using an EWS, in order for this to be contextualised with quantitative data from the observational study.

#### Recruitment and data collection

A combination of convenience and purposive sampling was used (16,17). The team developed a pre-defined sampling matrix with an aim to include opinions from a diverse group of mothers of hospitalised preterm newborn infants, their family members and health professionals. For mothers, this included their age and gestational age of their infant at birth and for health professionals, their professional discipline and level of experience. Mothers/family members were recruited via the three participating hospitals. Six focus group discussions (FGDs), involving 42 mothers/family members were conducted in parallel with quantitative data collection. Twenty-eight semi-structured interviews with health professionals, MoH commissioners and other stakeholders in newborn care were also held once quantitative data collection had completed. No further FGDs or interviews took place once data saturation was reached, defined as no new themes being generated during discussions nor creation of new codes or themes. Both the in-country and UK data analysts discussed the rolling data collection with the interviewing researcher and minor amendments to the schedule were made where necessary.

All FGDs and interviews were conducted by a female Kenyan researcher, based in Kenya, who had no prior relationship with participants. The researcher had no connection to the hospitals, was of a similar age, gender and social status as most of the mothers and we consider it unlikely that the mothers or the professionals would have been biased by social desirability bias. As the researcher had no connection to the hospitals and was not involved in any clinical care for babies or mothers, concern to please the researcher, and thus a potential power imbalance, would have been minimal. Participants’ confidentiality and anonymity was maintained by no real names being used and participants being identified with a simple code.

#### Data management and analysis

Data from focus groups and semi-structured interviews were conducted either in English or Swahili. All were recorded using a simple audio-recorder. Swahili interviews were translated and transcribed by the bilingual researcher and a selection of Swahili transcripts were given to the third analytic researcher (EO) to confirm accurate translation. Transcripts were stored on a university password-protected computer and shared with analysts via a secure link using Google Drive. No identifiable information was included on transcripts. Data were analysed using Thematic Network Analysis (18) after data cleaning and processing using NVIVO software (QSR international Pty Ltd.). The analysis was done using the framework of Thematic Network Analysis (18), a validated tool for building Global themes for theory generation from basic lexical and semantic coding of the transcripts in detail. Trustworthiness and reliability were examined at each step of the data collection and interpretation using Thematic Network Analysis (18). The authenticity of the data from the varied stakeholders was assessed by the comparison of interviews/FGDs; an expected amount of both agreement and contrast was noted. The analysis was subject to two experienced researchers (PP, HN) coding the data independently, before moving on to identify Organising Themes (18) in collaboration. The third researcher (EO) reviewed a sample of data and findings at each point of analysis for coherency and conformity. Further, the multi-site aspect of the study showed expected variation, depending on the location and the services offered, as well as confirming the Global themes which were applicably transferable to different hospital contexts.

## Results

### Quantitative data

**Fig. 1:**
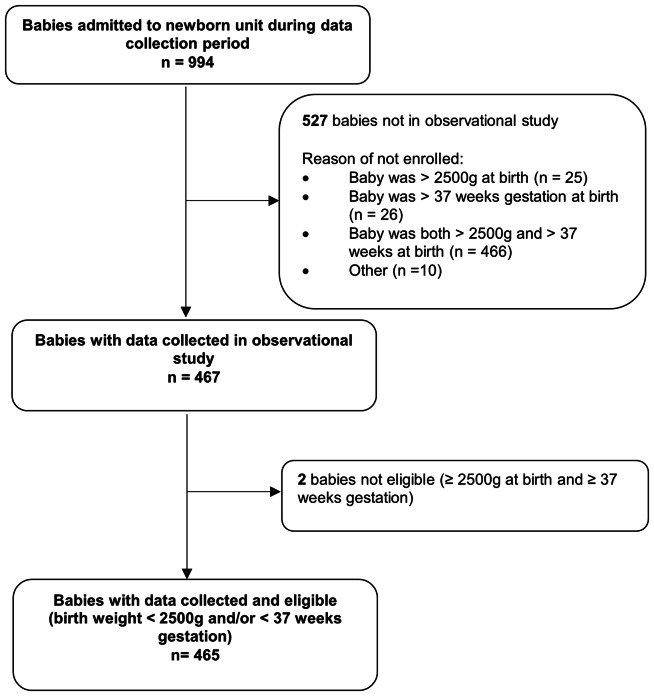
Study flowchart

During the collection period, 994 infants were admitted to the participating newborn units, of which 465 (47%) infants were eligible for the study (Fig. 1). The median birthweight was 1785 g (range: 430–3480 g) and the mean estimated gestational age (GA) at birth was 32.5 weeks (SD = 3.8).

The median time between birth and newborn unit admission was 2.2 hours (IQR: 1–4.4 h) and the main reason for admission was prematurity (260 infants, 56%) (Table 1).

**Table 1:** Baseline characteristics

	Total (*N* = 465) (%)
Gender	
Male	227 (49%)
Female	238 (51%)
**Place of birth**	
In this hospital	362 (78%)
In another hospital	82 (18%)
Home	21 (5%)
**Birthweight (grams)**	
Median [IQR]	1785 [1300, 2100]
Min, max	430, 3480
**Estimated gestational age at birth (completed weeks)**	
Mean [SD]	32.5 [3.8]
< 28 weeks	52 (11%)
28–32 weeks	142 (31%)
33–36 weeks	212 (46%)
≥ 37 weeks	48 (10%)
Not known	11 (2%)
**Methods of gestation age estimation**	
Early gestational ultrasound	19 (4%)
Mother’s last menstrual period	363 (78%)
Antenatal clinical examination of mother	6 (1%)
Clinical examination of baby	36 (8%)
How gestational age was calculated is unknown	30 (6%)
Gestational age at birth is unknown	11 (2%)
**Mode of delivery**	
Spontaneous vaginal	270 (58%)
Instrumental vaginal	1 (< 0.5%)
Emergency Caesarean delivery	187 (40%)
Elective Caesarean delivery	7 (2%)
**Time between birth and admission to newborn unit (hours)**	
Median [IQR]	2.2 [1, 4.4]
Min, max^1^	0, 943.8
missing	14* (3%)
**Main reason for admission**	
Prematurity	260 (56%)
Very low birth weight	26 (6%)
Surgical care	5 (1%)
Cardiac Care	2 (< 0.5%)
Respiratory disease	135 (29%)
Feeding support	2 (< 0.5%)
Infection	13 (3%)
Social care (unwell mother)	5 (1%)
Unknown	1 (< 0.5%)
Other	16 (3%)

1-The infant with the maximum value of time between birth and admission was born and admitted to the newborn unit at private wing of the hospital. The baby was later admitted to public newborn unit at the due to the limited resources in the private wing

* Missing values caused by 12 infants had time of birth missing; and 2 infants had admission time prior to time of birth

#### Clinical outcomes

By the end of the data collection period, 115 (25%) infants had died before discharge. The median age at death was 41.8 hours (IQR: 14.2–109.8 hours). 247 (53%) infants had been discharged home with median duration of hospital stay 9 nights (IQR: 4–18 nights), and 103 (22%) infants remained in hospital, at the end of the data collection period, with median length of hospital stay 9 nights (IQR: 4–23 nights).

#### Completion outcomes

Almost all eligible infants (*n* = 439, 94%) had the adapted NEWS-K CNMC form completed at least once during their hospital stay with a mean of 2.8 (SD 2.1) sets of vital signs recorded per infant per day (Table 2). Completion rates ranged between 81 and 99% between hospitals.

**Table 2:** NEWS-K number and timing of completion

	Total (*N* = 465)
NEWS-K CNMC completed at least once during infants hospital stay	
No	26 (6%)
Yes	439 (94%)
If no, reason for NEWS-K CNMC not completed	*n* = 26 (%)
Unknown	5 (22%)
Staffing shortages	17 (74%)
Other	1 (4%)
Missing	3 (12%)
**Percentage of days infant had NEWS-K CNMC completed**	
Median [IQR]	84.6 [50, 100]
Min, max	0, 100
0 – 49%	109 (23%)
50 − 74%	70 (15%)
75 − 89%	78 (17%)
90 – 100%	208 (45%)
**Average number of sets of vital sign observations per infant per day taken on NEWS-K CNMC**	
Mean [SD]	2.8 [2.1]

**Fig. 2:**
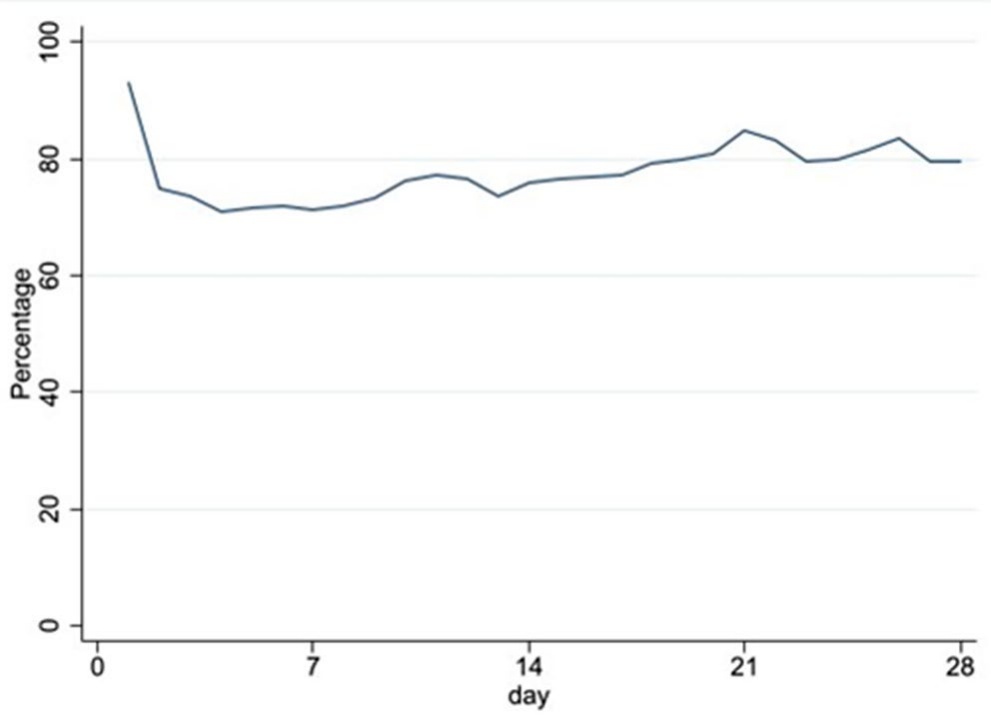
NEWS-K CNMC completion over 28 days

Completion of the NEWS-K CNMC was highest on day of admission to the newborn unit (93%), reducing to 75% by day 2. Over the neonatal period (i.e. 28 days), the percentage of infants with NEWS-K CNMC completed remained stable (Fig. 2).

#### Escalation outcomes

Table 3 shows the number of *sets* of vital sign observations, and the total *number* of observations, escalated to a senior staff member, in accordance with guidance. The percentage of abnormal observations escalated appropriately varied between sites (KNH, 11.2%; other two hospitals, 95%).

2652/7514 (35.3%) red or amber zone observations were due to a temperature outside of normal range. 2061/2652 (77.8%) of these observations were accompanied with a recorded temperature of *≤* 36.4 °C (i.e. hypothermia) of which 329/2061 (16%) were due to a temperature < 35.5 °C (i.e. moderate hypothermia).

**Table 3:** NEWS-K CNMC triggers and escalation

	Amber zone	Red zone
**Total number of sets of vital signs observations as recorded in daily data collection form**	14,987	
**Total number of sets of vital signs observations requiring escalation**	3984 (26.6%)	2039 (13.6%)
**Total number of vital sign observations requiring escalation as recorded on daily data collection/escalation form**	7514	
	4985 (66.3%)	2529 (33.7%)
**Issue escalated to a more senior staff member as per traffic light system on the NEWS-K CNMC form**		
No	3963 (79.5%)	1588 (62.8%)
Yes	572 (11.5%)	557 (22%)
Unknown*	450 (9%)	384 (15.2%)

*Unknown due to missing escalation form for the triggered vital sign

For each set of vital signs observations requiring escalation, the infant’s condition since the last set of observations was documented on the reverse of the NEWS-K CNMC form. This was completed for 4657 sets of vital signs observations requiring escalation in the red and/or amber zone. The infant’s condition improved for 1119 (24%), stayed the same for 1852 (40%), deteriorated for 578 (12%) and was unknown (due to the issue not being documented on the reverse of the NEWS-K CNMC) for 1108 (24%).

#### Feasibility indicators

Outcomes for demonstrating feasibility of using NEWS-K-CNMC forms are given in Table 4. 356/465 (76.6%) eligible infants had the forms completed on at least half of the days it should have been completed and 15% of vital signs requiring escalation were appropriately escalated.

The mean percentage of days the NEWS-K CNMC was completed during the infant’s admission was: morning shift (07:00-12.59), 55.3% (SD 36), afternoon (13:00–18:59), 49.6% (SD 35.2) and night (19:00–06:59), 56.6% (SD 38.1). Vital signs were escalated to a more senior member of the staff 20.4% of the time during a morning shift, 15.0% for an afternoon shift and 15.4% for a night shift.

**Table 4:** Summary of feasibility indicators

Indicator	Definition	Observed in NEWS-K (95% CI)
**NEWS-K CNMC form is completed**	Percentage of eligible infants who had vital signs recorded using the NEWS-K CNMC form on at least 50% of the days CNMC should have been completed	76.6% [72.4%, 80.3%]
**Escalation of care, according to the NEWS-K CNMC form**	Percentage of episodes when care was escalated where care was required to be escalated to a more senior member of staff in accordance with the NEWS-K CNMC form	15.0% [14.2%, 15.9%]
**Time of completion of the NEWS-K CNMC form**	Difference in % completion of the NEWS-K CNMC form and subsequent escalation of care between morning, afternoon and night	
*Difference in % completion of the NEWS-K CNMC form*		
Morning – afternoon		5.8 [3.6, 7.9]
Morning – night		-1.3 [-4.0, 1.4]
Afternoon- night		-7.0 [-9.5, -4.6]
*Difference in % subsequent escalation of the NEWS-K CNMC form*		
Morning – afternoon		5.4 [2.9, 7.9]
Morning – night		5.0 [2.9, 7.1]
Afternoon- night		-0.4 [-2.6, 1.8]

#### Staff in newborn unit

Staffing ratios are given in Table 5. Infant to nurse ratios were similar across the three shifts but there were fewer doctors during the afternoon and night shifts.

**Table 5:** Number of staff and infants in newborn unit

	Morning (7:00–12:59)	Afternoon (13:00–18:59)	Night (19:00–06:59)
Ratio between infants and nurses			
Mean [SD]	10.2 [1.7]	12.4 [1.6]	11.4 [1.5]
**Ratio between infants and doctors**			
Mean [SD]	16.7 [8.9]	47.8 [6.2]	53.8 [10.8]

#### Health professional experience of using the NEWS-K CNMC form

45 healthcare professionals completed the online questionnaire (Table 6) of which 35/45 (78%) reported using the NEWS-K CNMC form. Most healthcare professionals were general or neonatal nurses (29/45, 64%), with others either being paediatricians (*n* = 5, 11%) or other medical roles (e.g., medical officers) (*n* = 11, 24%). 33/35 (94%) who used the form said they liked using it for the reasons given in Table 6. 29/35 (83%) healthcare professionals identified challenges in implementing the forms in routine use. Most (29/35) stated that staff shortages were the main challenge.

**Table 6:** Healthcare professionals’ opinions of using NEWS-K CNMC form

	Total (*N* = 45)
Completed NEWS-K CNMC form for NEWS-K study	
Yes	35 (78%)
No	10 (22%)
**Liked using NEWS-K CNMC (n = 35)**	
Yes	33 (94%)
No	1 (3%)
Unsure	1 (3%)
n	35
Reason for liking the NEWS-K CNMC compared to the other CNMC* (not mutually exclusive)	
Gave me somewhere to record observations in an organised way	26 (74%)
Allowed me to see how the baby had been before I came on shift	30 (86%)
Helped me monitor baby better as I could easily see if escalation of care was needed	32 (91%)
Helped me communicate the baby’s condition to other professionals better	33 (94%)
Other	1 (3%)
Challenges of routinely using CNMC* (not mutually exclusive)	
We do not have enough staff to do this for every baby	29 (83%)
It requires colour printing	9 (26%)
When the form tells me to escalate the baby’s care, I am unable to do so because of lack of staff	13 (37%)
Other	2 (6%)

*Percentages used the number of health professionals completed NEWS-K CNMC during the study as denominator

### Qualitative data

Here we report only the facilitators and barriers of using the NEWS-K CNMC form. A deeper analysis of the context and communication in the dialectical and/or contested relationship between the stakeholders, including the parents and carers, will be reported separately. Three global themes, “rationale for NEWS-K CNMC form”, “implementation of NEWS-K CNMC form and “sustainability of NEWS-K CNMC form” were generated by the data (Table 7). A summary of qualitative study participants’ characteristics is given in supplementary material 5.

**Table 7:** Themes generated from qualitative data

Global Themes	Organising Themes	Example of a basic code for this organising theme
Rationale for NEWS-K CNMC form	Acceptable, enthusiastic staff	NEWS-K facilitators handover
	Easy to use	Frequency of monitoring
	Fits in with saving lives improvement work - TIME	Mothers doing basic observations
Implementation of NEWS-K CNMC form	NEWS-K CNMC as a SOP	Enables nurses to be proactive
	‘Sensitisation’	Policy makers different from doctors
	Education and training/systems and structures	Staff training, Staffing and colour printers
Sustainability of NEWS-K CNMC form	Use of NEWS-K CNMC form for research and audit	Improving documentation
	Tool works both ways	Triage using NEWS-K CNMC form
	Family centred care and education including community education	Understanding treatment plans

Overall, the NEWS-K CNMC was viewed positively. There was a clear rationale seen by both the clinicians and parents for the use of the tool, as this nurse discusses.

*“This chart has so many advantages because it gives (actually) it is all what a nurse and a doctor need for this baby to be monitored, all the vital observations that a baby needs they are there. I (KNH01)*.

Another key theme, with over 117 basic codes generated, was that of the sustainability of the implementation of the form, which was seen to be both desirable and possible. A doctor commented:

*“It would because it is something that is making work easy when you’re reviewing the baby. Considering the charts we were using there before, we were using an observation chart separately, we were using input output chart separately and then for the escalation we were using a nursing care plan separately. So, we were using three charts but this one now has taken care of the three charts. So, even in two years or three years’ time to come, I think it will still be in use. Yeah. Because it has combined everything. Yeah.” (KNH03)*.

However, barriers to using the NEWS-K CNMC were identified by both parents and health professionals. A key barrier was seen as staffing, and the need to sensitise staff to the new tool so that its use is more consistent across shifts – particularly among the night shift or agency and transferred staff.

*“Yeah, also we should have more of those trainings at least to refresh the staff. If we could get those refresher courses for the new nurses, I believe this can greatly improve the quality and the survival rates of these babies.” (MMLKH05)*.

The form was viewed as a tool that alerted staff and sometimes parents to the fact that the infants needed an intervention. The perceived response was often a need for action versus escalation if the infant’s observations were in the “red” zone, however, this was often impeded by the heavy workload for the nurses.

*“Like, we are usually so busy so you may find the nurse really wants to document everything done, action taken but it is not possible.” (TKH02)*.

*“I wish it can be rolled out to other facilities because now even with this the mothers were able to say, ‘I can say my baby has a temperature that is less than 35 and it is in the red zone. Does it mean my baby is in danger?’ So, even before you talk to the mother, the mother has already seen a warning sign.” (KNH03)*.

Finally, an interesting theme was that of the use of NEWS-K CNMC form to identify improving infants and transfer them to the stepdown kangaroo-care unit, which would improve their wellbeing (lower risk of infection, better bonding with care givers and breastfeeding) as well as freeing up space and time for more seriously unwell or deteriorating infants. This was seen as a huge positive and was much remarked on by both staff and parents, as this doctor explains:

*“Okay, you’re able to tell where your patient is. I don’t know how I would express it but it’s able to guide you on how to give care to your patient and also, you’re even able to categorize your patient in terms of who is the most sick and who is, who needs more attention, who maybe you can concentrate on and who maybe a baby who is out of danger and needs to be discharged [to step down Kangaroo care] ” (TKH07)*.

Most health professionals talked about the sustainability of the tool, some suggesting digital charts (though they recognised the many barriers to implementation), a black and white chart to avoid the need for colour printing or reusable charts which would reduce the need for printing.

*“Otherwise, if we had the coloured printers, we would have continued using it even after the study because people are already asking about it, what happened now that it’s no longer available. Yeah. The only challenge we have is we don’t have a coloured printer in the hospital and we do printing of our charts internally.” (TNH03)*.

Overall, in terms of implementation, sensitisation and training, the tool was found to be appropriate and potentially leading to a significant improvement in care.

The tool was also appreciated and commented on by the FGDs with mothers/family members, as it provided a simple way of communicating with staff and of parents being able to see quickly how the baby was doing and what care had taken place:

*Yes, to tell them what we want, I have heard you have said the doctors can do a note and leave it at the incubator where the baby is, what about you when you want to talk to the doctor what will enhance the communication (KNH01).*

However, the patchy nature of care was commented on in all FGDs, particularly that some staff were more communicative and inclusive than others.

*“Most of the time, it’s not easy because the things that worry us as mothers to them it is something easy or small. So, if you tell them with all the worries you have, you will still be told the baby is okay, it’s not in trouble. But you just can’t be satisfied with the answer you get. Even if you have an issue you just question why to go tell them and you’ll be told its okay or it’s not a big deal? What I would like is maybe for them to come and check the baby and then tell me it is normal or maybe if the symptoms persist I could come and report again. Something like that. That could make me feel better.” (TH01)*.

The parents and families’ main concern was that timely care be given, and although many agreed that the tool would help support this, staffing issues, particularly at night were identified as a major problem to delivering timely care, even if it were better identified using the tool:

“*Finding them isn’t hard especially during day time but at night sometimes you will find them asleep and we are left alone but during day time it is very easy to find them.” (TH01)*.

## Discussion

This is the first study to report the use of a neonatal EWS in Kenya, in the context of a feasibility and acceptability study to inform the design of a pragmatic randomised clinical trial to evaluate its effectiveness in improving neonatal outcomes. One of the major challenges faced by neonatal services in LMICs is the shortage of staff, which is well documented (6–8, 19) and confirmed by the poor infant to staff ratios we report in Table 5, and qualitative data from health professionals and parents. As described in the qualitative study theme “Implementation of the NEWS-K CNMC form”, in addition to the need for adequate staffing levels for the use of an EWS, ensuring staff are in place for training and sensitisation is also important. Introduction of a monitoring tool that increases workload without adequate evidence to show that its use improves outcomes may be counterproductive hence an adequately powered, high-quality randomised trial is vital before routine use of EWS is recommended as routine practice in LMIC settings.

Although a newborn monitoring chart had begun to be implemented in some Kenyan hospitals (Comprehensive Newborn Monitoring Chart; CNMC), crucially the charts do not include the key EWS feature; the traffic-light system. Adapting the CNMC to include this feature was an important component of our study and enabled health professionals to be visually alerted when vital signs were abnormal, i.e., in a red or amber zone. The chart changed from a monitoring chart whereby vital signs were *tracked*, to one which triggers action, and health professionals reported liking the fact the EWS combines everything in a single form. However, data from the qualitative interviews and survey of health professionals demonstrated that the colour-based nature of the chart did present some challenges, particularly in relation to colour printing in a low-resource setting. In the future, consideration should be given to reusable or digital charts, though both options also present different challenges that would need further exploration. The unique nature of the EWS visually alerting health professionals and parents to when action is needed, could also help to prioritise care which, in a low resource setting, could be important, enabling the right care to be given at the right time. Health professionals could more easily target care to those infants who need it similarly enabling the babies who are less sick to receive less interventions.

We found that it may be possible to conduct a clinical trial to evaluate the use of the EWS. We met our pre-specified feasibility criteria in two of three domains. We found that it is possible to use the chart to record vital signs. More than three quarters of eligible infants had the charts completed on at least 50% of the days that the charts should have been completed. This was slightly higher than our feasibility cut-off of 75% (supplementary material 3) of the infants though health professionals recognised that despite their best intentions of completing the form, sometimes this was not possible due to their heavy workload. Given that poor record keeping is a known problem in this setting(20, 21) this is an important finding. The qualitative data also confirmed that form completion also facilitates standardised, visual two-way communication between health professionals and parents, which was seen as a benefit and aligned with a more family-centred care approach. For parents, this is seen as particularly important when infants whose vital signs may be tracked on the amber or red zones, where concerns may be raised. It would be important to educate parents about what the chart indicates and how the chart should be used.

We found no important differences in percentage completion of the chart on different shifts, meeting our pre-specified feasibility criteria. As expected, a higher number of observations were recorded at night as this shift was twice as long as the morning and afternoon shifts. Shift periods were selected to match the participating newborn units nursing shift times.

Our final feasibility criterion was the percentage of time that care was escalated to a more senior member of staff in accordance with the specifications on the chart. We found that this occurred only on 15% of episodes where a vital sign in the red or amber zone was recorded. This was below the pre-specified feasibility cut-off of 40%. There was substantial variation between the participating newborn units, though there could be some explanations for this. Firstly, the differences in infant to staff ratios and, secondly, the differences in team structures within each newborn unit, e.g. in a newborn unit with a more hierarchical structure, it could be less likely for the nurse to request a senior review (22). In addition, the Kenyatta National Referral hospital is much larger than the other participating hospitals, has a larger team of clinical staff which can make implementation more challenging, and, by its nature of being a referral hospital, often cares for sicker newborns. The traffic-light system that was added to the CNMC chart, is adapted from NEWTT, a tool developed in the UK (23), a high-income country, where the health system is very different with a higher staff to patient ratio and evolving health care team dynamics that empower all cadre of staff to escalate health care concerns with a requirement that senior staff respond in a timely manner. If a vital sign was plotted in the red or amber zones, the chart specifically stated, “escalation to doctor”. However, in the hospitals participating in this study this is not always possible due to the well-documented extreme staff shortages previously described, and, in many cases, appropriate action could be taken by a member of nursing staff. Qualitative data showed that health professionals also preferred the terminology of ‘action’ rather than ‘escalation’, and this would be an important adaptation to make in the future, reflecting that many of the components of essential newborn care can be appropriately undertaken by nursing staff, without involvement of a doctor. The issue of lack of staffing was a central theme within the qualitative data, with health professionals recognising the need for having appropriate staffing in place to implement interventions, directed by the NEWS-K CNMC, but also recognised the importance of training, to increase adherence to the ‘triggering’ component of the NEWS-K CNMC. Providing ongoing, refresher training would be important an important part of sustainability and implementation in the future.

Of notable clinical importance was the fact that many infants in the study were cold, with many instances of temperatures of < 35.5°c being recorded, i.e. in the ‘red zone’ requiring escalation. This has been a key finding in other observational studies in Kenya, particularly in busy tertiary referral hospitals (24). Given the well-documented association between temperature and mortality (25), an EWS has the potential to identify hypothermic infants sooner, leading to evidence-based interventions known to increase temperature being implemented, such as Kangaroo Mother Care.

### Strengths and limitations

Our observational study included infants and health professionals from three hospitals representing different levels of neonatal care and within different levels of the Kenyan health system, although we acknowledge all hospitals were located in just two Kenyan counties (Nairobi County and Kiambu County). We aimed to collect data on 432 eligible infants and exceeded this slightly (*n* = 465), due to a slight extension in the data collection period, thus giving us an adequate sample on which to assess feasibility. We have also included the views and opinions of health professionals and parents in the development of a neonatal early warning score in this setting.

However, we recognise there are some limitations of the study design. Firstly, we were only able to collect data for the duration of the infant’s neonatal stay, and therefore do not know the infant outcomes post-discharge. In addition, data was collected on whether the action taken was an appropriate clinical response in line with the hospitals’ local guidelines, however we were unable to determine whether the action taken led to an improvement in the infant’s condition. Funding for the study was limited to twelve months only, leading to ambitious study timelines, and restricting longer periods of time for training etc. It would have been beneficial to have had an implementation period prior to data collection. This would have enabled health professionals to adapt to using the form, taking action on vital signs when required, which could have resulted in “hitting the ground running” and potentially higher quality of data collection. Finally, in the context of determining feasibility for a randomised trial, we did not collect data on the number of newborn units who would be willing to participate, thus cannot currently determine whether an adequately powered trial would be possible. From a study-conduct perspective, we also experienced several challenges. Of note, the study was disrupted part way through due to UK funding uncertainties in global health research. This led to activity being paused temporarily, thus potentially losing momentum and “buy-in” within the newborn units, which could have subsequently affected data collection.

Overall, we found that it is feasible to *track* vital signs using a standard monitoring chart. However, feasibility has not been demonstrated in *triggering action* when an abnormal vital sign has been documented. This could be for several reasons previously outlined, including terminology used on the form and/or staffing issues. Families and health professionals support the use of an EWS on newborn units and like its simplicity and that it supports communication, facilitating family-centred care. However, any tool or intervention implemented in this setting must consider system-level issues, in particular inadequate and under-resourced staffing levels in newborn units.

## Conclusion

Our data shows that there is value in continuing to explore the value of a neonatal EWS in this setting, however further adaptations to the CNMC form would be required before further evaluation. In order to evaluate whether an EWS could improve clinical outcomes, including reduction in mortality, a pragmatic randomised clinical trial is needed.

### Electronic supplementary material

Below is the link to the electronic supplementary material.

Supplementary Material 1

Supplementary Material 2

Supplementary Material 3

Supplementary Material 4

Supplementary Material 5

## Data Availability

Data is available via the Nottingham Research Data Management Repository and can be accessed here: https://rdmc.nottingham.ac.uk/handle/internal/10509.
