# Supplementary material for: A mixed-methods study to investigate feasibility and acceptability of an early warning score for preterm infants in neonatal units in Kenya: results of the NEWS-K study: Neonatal early warning scores in Kenya
Source: BMC Pediatr. 2024 May 11;24:326. doi: 10.1186/s12887-024-04778-z (PMC11088162; doi:10.1186/s12887-024-04778-z)
Supplement: Supplementary file 1 — Supplementary Material 1 [file 12887_2024_4778_MOESM1_ESM.pdf]

NEWS-K Healthcare Professional's views

Introduction

You have been sent this short questionnaire because you work on the Newborn Unit of a hospital participating in the NEWS-K study, which is investigating the feasibility of using a neonatal early warning score in a low resource country such as Kenya.

The questionnaire will take approximately 5-10 minutes to complete and your answers will help us understand your views on the NEWS-K CNMC form.

Your answers are completely anonymous.

Thank you for your time.

Question 1

Have you worked on the Newborn Unit at Kenyatta National Hospital, Mama Lucy Kibaki Hospital or Thika County Hospital between **19th July 2021 and 17th September 2021?**

✱ *Required*

- ◯ Yes
- ◯ No

Question 2

In which Newborn Unit do you work?

- Kenyatta National Hospital
- Mama Lucy Kibaki Hospital
- Thika County Hospital
- None of the above

Question 3

What is your role?

- Paediatrician
- Neonatologist
- Neonatal nurse
- Nurse
- Other

If you selected Other, please specify:

How long have you worked in the newborn unit (please give to nearest year)?

Before the NEWS-K study, had you used the CNMC form to monitor babies?

- Yes
- No
- Unsure

Question 4

Where did you use the CNMC previously?

Question 5

Between **19th July 2021 and 17th September 2021,** did you complete the revised CNMC form (NEWS-K CNMC form) for the purpose of the NEWS-K study?

- Yes
- No

Question 6

If you did not complete the CNMC form, why not?

- [ ] Form wasn’t available for completion
- [ ] Didn’t realise I needed to
- [ ] Hadn’t been trained in how to complete the form
- [ ] My supervisor/manager told me not too
- [ ] Lack of time
- [ ] Other

If you selected Other, please specify:

Question 7

Did you like using the NEWS-K CNMC form?

- Yes
- No
- Unsure

Question 8

Compared to the standard CNMC form, what did you like about the form? Tick as many as apply and give other reasons in the box provided.

- ☐ Gave me somewhere to record observations in an organised way
- ☐ Allowed me to see how the baby had been before I came on shift
- ☐ Helped me monitor baby better as I could easily see if escalation of care was needed
- ☐ Helped me communicate the baby’s condition to other professionals better
- ☐ Other

If you selected Other, please specify:

Question 9

What did you dislike about the form? Tick as many as apply and give other reasons in the box provided.

- ☐ It created extra work without much benefit to the patient
- ☐ I did not know what to do when escalation of care was recommended
- ☐ We do not have enough staff to do this extra work
- ☐ Other

If you selected Other, please specify:

Question 10

In your opinion, what are the challenges to routine use of such forms in your hospital? Tick as many as apply and give other reasons in the box provided.

- ☐ We do not have enough staff to do this for every baby
- ☐ It requires colour printing
- ☐ When the form tells me to escalate the baby's care, I am unable to do so because of lack of staff
- ☐ Other

If you selected Other, please specify:

Question 11

Is there anything you would change about the NEWS-K CNMC form in the future?

- Yes
- No

Question 12

What would you change?

Question 13

If you have anything else you'd like to add about the NEWS-K CNMC form, the study in general or neonatal early warning scores in general, please do so here.

Thank you

You have now completed the questionnaire. Thank you very much. If you have any questions, please email news-k@nottingham.ac.uk
