# Supplementary material for: A mixed-methods study to investigate feasibility and acceptability of an early warning score for preterm infants in neonatal units in Kenya: results of the NEWS-K study: Neonatal early warning scores in Kenya
Source: BMC Pediatr. 2024 May 11;24:326. doi: 10.1186/s12887-024-04778-z (PMC11088162; doi:10.1186/s12887-024-04778-z)
Supplement: Supplementary file 2 — Supplementary Material 2 [file 12887_2024_4778_MOESM2_ESM.pdf]

Study ID: ___ ___ - ___ ___ ___ ___

NEWS-K CNMC FOR USE IN NEWS-K STUDY ONLY Version 1.1 04 Feb 2021

Name	IP NO	Sex M □ F □	D.O.A	D.O.B
Date today	Diagnosis			
Birth Wt gm	*Interventions: CPAP □ Oxygen □ Phototherapy □ Blood tranfusion □ Exchange transfusion □ KMC □*			

Daily Clinician Feed and Fluid prescription

Day of Life	Current Wt = gm

Total input(feed and fluid) 24hrs = ml

Feed: BF ☐ EBM ☐ Term Formula ☐ Pre-Term Formula ☐

Route: Cup☐ NGT☐ OGT☐

Volume & Frequency = _________ml 3hrly ☐ 2hrly ☐

Total 24hr Volume = _________ml

IV Fluid & Additives	Vol (ml)	Duration

Other prescribing instructions

Clinician's name Time:

Daily IV Fluid Nursing plan

Start time:

Hourly rate= _________ ml (____ drops/min)

Planned vol = _________ ml in_______ hrs

	Monitoring Freq___hrs | Time												
Vitals	Temp (^{0}C)												
	Pulse (b/min)												
	Resp Rate (b/min)												
	Oxy Sat (%) or Cy^{0} Cy^{+}												
Assessment	Resp Distress 0,+,+++												
	CPAP Pressure (cm H_2O)												
	FiO_2 (%)												
	Jaundice 0,+,+++												
	Apnoea Y/N												
	Blood Sugar (mmol/l)												
	Completed by (name)												
Feed	Breastfeeding sufficient Y/N												
	EBM vol given (ml)												
	Formula vol given (ml)												
Fluid	IV volume given (ml)												
	IV Line working Y/N												
Output	Vomit Y/N												
	Urine Y/N												
	Stool Y/N												
	Completed by (name)												

Morning shift notes
Category: A□ B□ C□
Total feed+fluid in this shift_______ ml
Deficit_______ ml
Completed by (name)

Afternoon shift notes
Category: A□ B□ C□
Total feed+fluid in this shift_______ ml
Deficit_______ ml
Completed by (name)

Night shift notes
Category: A□ B□ C□
Total feed+fluid in this shift_______ ml
Total feed+fluid in 24hrs_______ ml
Deficit_______ ml
Completed by (name)

Jaundice 0 none, +mild(face),+++severe(feet)

Tick the category of baby after assessment

Alerts : circle readings outside normal range with red pen and action. SEE REVERSE

Time:	Issue: e.g. Temp 35.9	Dr's Initials:	Action taken: Describe plan and action taken	Infants condition: (since last set of obs)
				Improved: ☐<br>Stayed the same: ☐<br>Deteriorated: ☐
				Improved: ☐<br>Stayed the same: ☐<br>Deteriorated: ☐
				Improved: ☐<br>Stayed the same: ☐<br>Deteriorated: ☐
				Improved: ☐<br>Stayed the same: ☐<br>Deteriorated: ☐
				Improved: ☐<br>Stayed the same: ☐<br>Deteriorated: ☐
				Improved: ☐<br>Stayed the same: ☐<br>Deteriorated: ☐
				Improved: ☐<br>Stayed the same: ☐<br>Deteriorated: ☐
				Improved: ☐<br>Stayed the same: ☐<br>Deteriorated: ☐
				Improved: ☐<br>Stayed the same: ☐<br>Deteriorated: ☐

ACTION:	Immediate escalation to Dr	IF 1: Escalate concern to senior and repeat in 30 mins<br>IF +1: Immediate escalation to Dr	Continue obs as planned	IF 1: Escalate concern to senior and repeat in 30 mins<br>IF +1: Immediate escalation to Dr	Immediate escalation to Dr
Temperature:	Less than 35.5	35.6 – 36.4	36.5 – 37.5		Greater than 37.5
Pulse:	Less than 80	80 – 99	100 – 160	161 – 180	Greater than 180
Resp Rate:	Less than 30	30 – 39	40 – 60	61 – 80	Greater than 80
O2 Sats on O2:	Less than 90%		90 – 95%		Greater than 95%
On room air:	Less than 90%	90 – 94%	95 – 100%		
Blood sugar:	Less than 1.9	1.9 – 2.5	2.6 – 6.6	6.7 – 9.9	Greater than 10
