# Supplementary material for: A mixed-methods study to investigate feasibility and acceptability of an early warning score for preterm infants in neonatal units in Kenya: results of the NEWS-K study: Neonatal early warning scores in Kenya
Source: BMC Pediatr. 2024 May 11;24:326. doi: 10.1186/s12887-024-04778-z (PMC11088162; doi:10.1186/s12887-024-04778-z)
Supplement: Supplementary file 3 — Supplementary Material 3 [file 12887_2024_4778_MOESM3_ESM.docx]

**Supplementary Material 3: Pre-specified feasibility criteria**

| **Indicator** | **RED** | **AMBER** | **GREEN** |
| --- | --- | --- | --- |
| 1. **NEWS-K CNMC form is completed** | **<60%** of eligible infants had vital signs recorded using the NEWS-K CNMC form on at least **50%** of  the days CNMC should have been completed | **>60%** - < 75% of eligible infants had vital signs recorded using the NEWS-K CNMC form on at least **50%** of the  days CNMC should have been completed | **>75%** of eligible infants had vital signs recorded using the NEWS-K CNMC form on at least **50%** of the  days CNMC should have been completed |
| 1. **Escalation of care, according to the NEWS-K CNMC form** | Care was escalated **<40%** of the time where care was required to be escalated to a more senior member of staff in accordance with the NEWS-K CNMC form | Care was escalated **>40% - <60%** of the time where care was required to be escalated to a more senior member of staff in accordance with the NEWS-K CNMC form | Care was escalated **>60%** of the time where care was required to be escalated to a more senior member of staff in accordance with the NEWS-K CNMC form |
| 1. **Time of completion of the NEWS-K CNMC form** | Completion of the NEWS-K CNMC form and subsequent escalation of care (as required) very dependent upon the time of day; defined as **>75%** difference between completion in morning, afternoon and night | Completion of the NEWS-K CNMC form and subsequent escalation of care (as required) dependent upon the time of day; defined as >50% - **<75%** difference between completion in morning, afternoon and night | Completion of the NEWS-K CNMC form and subsequent escalation of care (as required) is not dependent upon the time of day; defined as **<50%** difference between completion in morning, afternoon and night |
