# Supplementary material for: A mixed-methods study to investigate feasibility and acceptability of an early warning score for preterm infants in neonatal units in Kenya: results of the NEWS-K study: Neonatal early warning scores in Kenya
Source: BMC Pediatr. 2024 May 11;24:326. doi: 10.1186/s12887-024-04778-z (PMC11088162; doi:10.1186/s12887-024-04778-z)
Supplement: Supplementary file 4 — Supplementary Material 4 [file 12887_2024_4778_MOESM4_ESM.docx]

**SUPPLEMENTARY MATERIAL 5 – Summary of qualitative study participants**

**Focus group participants (mothers and family members)**

|  | Total n=42 |
| --- | --- |
| Mothers  Mother age <20  Mother age 20-30  Mother age 30-40  Gestational age of baby of mothers  <28 weeks  <28^+0^-32^+6^  33^+0^-36^+6^ | 22  1  17  4  9  8  5 |
| Other family/support members  Spouse / husband / Father  Sister  Auntie  Friend  In-law  Unrecorded | 20  9  1  1  1  1  7 |

**1:1 interview participants (health professionals and other stakeholders)**

|  | n=28 |
| --- | --- |
| Role  Trained general nurse  Hospital manager  Neonatologist/paediatrician  Trained neonatal nurse  Trained paediatric nurse  Medical/clinical officer (doctor)  Government representative | 6  6  4  4  4  3  1 |
| Experience/seniority  Early career (qualified <5 years)  Mid/Senior career (qualified >5 years)  Not stated | 10  17  1 |
