# Supplementary material for: A mixed-methods study to investigate feasibility and acceptability of an early warning score for preterm infants in neonatal units in Kenya: results of the NEWS-K study: Neonatal early warning scores in Kenya
Source: BMC Pediatr. 2024 May 11;24:326. doi: 10.1186/s12887-024-04778-z (PMC11088162; doi:10.1186/s12887-024-04778-z)
Supplement: Supplementary file 5 — Supplementary Material 5 [file 12887_2024_4778_MOESM5_ESM.docx]

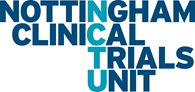


The feasibility and acceptability of an early warning score

for preterm and low-birth weight infants in Kenya

Statistical Analysis Plan for study 1

Final Version 1.0

21 JAN 2022

Based on Protocol version 1.0 (dated 04 Jan 2021)

| The following people have reviewed the Statistical Analysis Plan and are in agreement with the contents | | | | |
| --- | --- | --- | --- | --- |
| **Name** | **Job title** | **Trial Role** | Signature | Date |
| Yuanfei Su | Medical statistician | Trial Statistician (Author) | 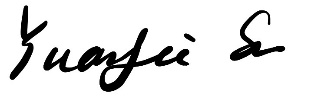 | 21Jan2022 |
|  |  |  |  |  |
| Lucy Bradshaw | Medical Statistician | Senior Trial Statistician | 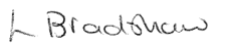 | 21Jan2022 |
|  |  |  |  |  |
| Eleanor Mitchell | Associate Professor of Clinical Trials | Chief Investigator | 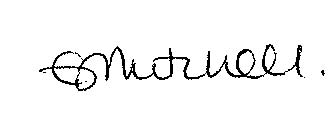 | 21 January 2022 |

Table of Contents

[**1.** **INTRODUCTION & PURPOSE** 5](#_Toc93648273)

[**2.** **SYNOPSIS OF STUDY DESIGN AND PROCEDURES** 6](#_Toc93648274)

[**2.1.** **Sample size and justification** 6](#_Toc93648275)

[**2.2.** **Blinding and breaking of blind** 7](#_Toc93648276)

[**2.3.** **Study committees** 7](#_Toc93648277)

[**2.4.** **Outcome measures** 7](#_Toc93648278)

[**3.** **INTERIM ANALYSIS** 9](#_Toc93648279)

[**4.** **GENERAL ANALYSIS CONSIDERATIONS** 9](#_Toc93648280)

[**4.1.** **Analysis sets** 9](#_Toc93648281)

[**4.2.** **Timing of final analysis** 9](#_Toc93648282)

[**4.3.** **Statistical software** 9](#_Toc93648283)

[**4.4.** **Derived variables** 9](#_Toc93648284)

[**4.5.** **Procedures for missing data** 13](#_Toc93648285)

[**5.** **DESCRIPTION OF PARTICIPANT CHARACTERISTICS** 13](#_Toc93648286)

[**5.1.** **Participant flow** 13](#_Toc93648287)

[**5.2.** **Baseline characteristics** 13](#_Toc93648288)

[**5.3.** **Infant outcome** 13](#_Toc93648289)

[**6.** **ANALYSIS OF FEASIBILITY OF NEWS-K** 14](#_Toc93648290)

[**7.** **ANALYSIS OF EFFECTIVENESS/EFFICACY** 15](#_Toc93648291)

[**8.** **HEALTHCARE PROFESSIONAL EXPERIENCE ANALYSIS** 15](#_Toc93648292)

[**9.** **FINAL REPORT TABLES AND FIGURES** 16](#_Toc93648293)

[**10.** **REFERENCES** 16](#_Toc93648294)

**Abbreviations**

| **Abbreviation** | **Description** |
| --- | --- |
| SAP | Statistical Analysis Plan |
| CNMC | Comprehensive Newborn Monitoring Chart |
| EWS | Early Warning Score |
|  |  |
|  |  |
|  |  |
|  |  |
|  |  |

**Changes from protocol**

The table below details changes to the planned analyses in the SAP compared to the protocol which after discussion with the TMG are not considered to require a protocol amendment.

| **Protocol version**  **and section** | **Protocol text** | **SAP version**  **and section** | **SAP text** | **Justification** |
| --- | --- | --- | --- | --- |
|  |  |  |  |  |

**Amendments to versions**

| **Version** | **Date** | **Change/comment** | **Statistician** |
| --- | --- | --- | --- |
|  |  |  |  |
|  |  |  |  |
|  |  |  |  |
|  |  |  |  |
|  |  |  |  |

**Additional contributors to the SAP (non-signatory)**

| **Name** | **Trial role** | **Job Title** | **Affiliation** |
| --- | --- | --- | --- |
|  |  |  |  |

1. **INTRODUCTION & PURPOSE**

This document details the rules proposed and the presentation that will be followed, as closely as possible, when analysing and reporting the main results from the prospective observational study of the Neonatal Early Warning Scores in Kenya project funded by Medical Research Council (MRC) Global Maternal and Neonatal Health Seed Funding.

The purpose of the plan is to:

1. Ensure that the analysis is appropriate for the aims of the study, reflects good statistical practice, and that interpretation of a priori and post hoc analyses respectively is appropriate.
2. Explain in detail how the data will be handled and analysed to enable others to perform or replicate these analyses

Additional exploratory or auxiliary analyses of data not specified in the protocol may be included in this analysis plan.

This analysis plan will be made available if required by journal editors or referees when the main papers are submitted for publication. Additional analyses suggested by reviewers or editors will be performed if considered appropriate. This should be documented in a file note.

Amendments to the statistical analysis plan will be described and justified in the final report of the study and where appropriate in publications arising from the analysis.

1. **SYNOPSIS OF STUDY DESIGN AND PROCEDURES**

| Title | The feasibility and acceptability of an early warning score for preterm and low-birth weight infants in Kenya |
| --- | --- |
| Acronym | NEWS-K study |
| Short title | Neonatal Early Warning Scores in Kenya |
| Chief Investigator | Eleanor Mitchell, Associate Professor of Clinical Trials |
| Objectives | To test the feasibility of implementing an early warning score system  for preterm and low-birth weight infants in neonatal units in Kenya, by  undertaking a prospective observational study |
| Study Configuration | Multi-centre mixed-methods study |
| Setting | Newborn Units in three hospitals in Kenya |
| Sample size estimate | No formal sample size has been calculated. The numbers of participants expected in the observational study is based upon the number of preterm infants expected to be admitted to the participating newborn units during the data collection period. |
| Number of participants | Anticipate data collection on ~432 infants and 24-30 healthcare professionals |
| Eligibility criteria | All preterm infants (defined as <37 weeks and/or <2.5kg) who are an in-patient in the participating in newborn units. “Inborn” and “outborn” infants can be included. |
| Description of interventions | Not applicable as there is no intervention in this study |
| Randomisation and blinding | Not applicable |
| Outcome measures | Outcomes will be measured for the study are to assess the feasibility and acceptability of completing the ‘NEWS-K CNMC’ form. |

- 1. **Sample size and justification**

For the observational study, data collection was planned on all eligible infants over a 6-week period. Using data from a previous study conducted at Kenyatta National Hospital and routine clinical data from Mama Lucy Kibaki and Thika County Hospital, we anticipated collecting data on around 432 infants during this time period, as demonstrated in the table below. The protocol acknowledged, however, that this number may alter depending on the global Covid-19 pandemic situation at the time of data collection. 432 infants will allow estimation of percentages with a margin of error (half-width of 95% confidence interval) of around 5 percentage points.

Table 1: Estimated number of eligible infants during observational study data collection period

| **Hospital** | **Estimated number of births (6 weeks)** | **Estimated number of eligible infants (6 weeks)** |
| --- | --- | --- |
| Kenyatta National Hospital | 1510 | 220 |
| Mama Lucy Hospital | 1446 | 149 |
| Thika Hospital | 1200 | 63 |
| **Total** | 4156 | 432 |

- 1. **Blinding and breaking of blind**

Not applicable

- 1. **Study committees**

The Chief Investigator has overall responsibility for the study and shall oversee all study management. The study will be managed on a day-to-day basis by a study coordinator based in the UK who will work closely with the Chief Investigator and other members of the study team, including the data coordinator, statistician, and co-investigators. The UK study coordinator will also liaise regularly with the Kenya study coordinator who is responsible for the coordination of the study at the three participating hospitals in Kenya and is based at the Kenyatta National Hospital. Whilst the Kenya study coordinator will have overall responsibility for study coordination in Kenya, they will liaise with the two coordinators based at the other two participating hospitals (Mama Lucy Kibaki Hospital and Thika County Hospital).

The co-investigators will form a study management group that will meet at regular intervals for the duration of the study. These meetings will be held using video conferencing facilities and be convened at times mutually convenient for all participating co-investigators, considering the time difference between the UK and Kenya. The UK study coordinator is responsible for the study management group meetings and will take minutes. The study coordinators based in Kenya will also attend study management group meetings.

The data custodian will be the Chief Investigator.

There is no independent study steering committee or data monitoring committee for this study.

- 1. **Outcome measures**

Outcomes for the study are to assess the feasibility and acceptability of completing the ‘NEWS-K CNMC’ form and are described in Table 1 below.

**Table 1:** Summary of the outcome measures

| **Completion outcome** | **Description** |
| --- | --- |
| NEWS-K CNMC completion summary | Including whether NEWS-K CNMC completed as protocol during infants hospital stay; and whether NEWS-K CNMC completed at least once during infants hospital stay. |
| Number of NEWS-K CNMC completion | Including number of days infant had NEWS-K CNMC completed; and number of days infant should have NEWS-K CNMC completed during hospital stay. |
| Timing of NEWS-K CNMC completion | Number of days infant had observations on NEWS-K CNMC during morning/afternoon/night. |
| NEWS-K CNMC completion according to level of care | Whether NEWS-K CNMC had been completed will be summarised by level of care. |
| Number of vital sign observations | Average number of vital sign observations per infant per day taken on NEWS-K CNMC. |
| Timing of vital sign observations | Average number of vital signs observations per infant per day taken on NEWS-K CNMC during morning/afternoon/night. |
| NEWS-K CNMC non-completion summary | Total number of days and reason NEWS-K CNMC not completed. |

| **Trigger and action outcome** | **Description** |
| --- | --- |
| NEWS-K CNMC triggers and escalation by site | Including total number of vital sign observations requiring escalation in amber/red zone, and whether issue had been escalated to a more senior staff |
| NEWS-K CNMC triggers and escalation by time of day | Total number of vital sign observations requiring escalation in amber/red zone during morning/afternoon/night |
| NEWS-K CNMC triggers, escalation, and action^1^ by vital sign | Total number of *xx* observations requiring escalation in amber/red zone where *xx* =:   - Temperature - Pulse - Respiratory - O2 sats (on O2) - O2 sats (on air) - Blood sugar |

1-whether clinical response was taken will be derived from the details of the escalation entered in the escalation and outcome form.

1. **INTERIM ANALYSIS**

Not applicable.

1. **GENERAL ANALYSIS CONSIDERATIONS**
   1. **Analysis sets**

The analysis sets for the prospective observational study will be infants born less than 37 weeks gestation and/or weighing less than 2500 grams at birth and who are in-patients on the newborn units of Kenyatta National Hospital, Mama Lucy Kibaki, and Thika Country Hospital between 19^th^ July 2021 and 17^th^ September 2021 (9 weeks, the data collection period was extended from the 6 weeks originally planned due to lower than expected number of babies).

| **Outcome** | **Analysis set** |
| --- | --- |
| Infant outcomes / Completion outcome / Trigger and action outcomes | All eligible infants (<37 weeks gestation and/or <2500 g) with data collected during the observational study. |

- 1. **Timing of final analysis**

Analyses of the prospective observational study will be conducted after database lock.

- 1. **Statistical software**

Analyses will be performed using Stata version 17.0 or above.

- 1. **Derived variables**

| **Variable** | **Derivation** |
| --- | --- |
| NEWS-K CNMC completed as protocol during infants hospital stay | This will be derived from the binary indicator – “was the NEWS-K CNMC completed today?” and the reason of non-completion on daily data collection form.  The value of the variable will be ‘Yes’ if the binary indicator was completed as ‘Yes’ or the reason of non-completion was ‘Clinical decision’ / ‘Baby in skin-to-skin care’ every day during infant hospital stay.  The value will be ‘Not required’ if the reason of non-completion, ‘Clinical decision’ or ‘Baby in skin-to-skin care’, was ticked every day during infant hospital stay.  Otherwise, the value of the variable will be ‘No’. |
| NEWS-K CNMC completed at least once during infants hospital stay | This will be derived from the binary indicator – “was the NEWS-K CNMC completed today?” and the reason of non-completion on daily data collection form.  The value of the variable will be ‘Yes’ if binary indicator is completed as ‘Yes’ at least once during the infant hospital stay.  If the reason of non-completion was ‘Clinical decision’ or ‘Baby in skin-to-skin care’ for all the days during infant hospital stay, the value of the variable will be not required.  Otherwise, the value of the variable will be ‘No’. |
| Total number of days NEWS-K CNMC not completed | The sum of the number of times Yes/No was ticked for the question “was the NEWS-K CNMC completed today?” on the daily data collection form. |
| Total number of days the completion of NEWS-K CNMC unknown | The sum of the differences between the number of infant’s daily data collection forms and the number of daily data collection forms that infant should have during hospital stay. |
| Total number of days NEWS-K CNMC not required | The sum of the number of times ‘Clinical decision’ or  ‘Baby in skin-to-skin care’ was ticked for the reason why CNMC was not completed today on the daily data collection form. |
| Number of days infant should have NEWS-K CNMC completed during hospital stay | Derived from the date of infant being admitted to the newborn units (enrolment form), date of death (death form), date of discharge (discharge form), and the date of the last daily data collection (17^th^ September 2021) as well as the reason NEWS-K CNMC not completed (daily data form)  The number of days not-required will be the sum of the days the reason of CNMC not completed was ‘Clinical decision’ or ‘Baby in skin-to-skin care’.  In addition, NEWS-K CNMC needs to be completed on the first and last day of the infant being in the newborn unit. An extra day will be added for the infants who were admitted to the newborn unit during 00:00 – 06:59 on the given day since vital signs observations between 00:00 and 06:59 were collected on the daily data collection form for the previous day.  - For infants who died prior to discharge,   1. death time during 00:00 – 06:59, this will be derived as date of death – the date of admission – the days not-required. 2. death time during 07:00 – 23:59, this will be derived as date of death – the date of admission + 1 – the days not-required.   - For infants who were discharged home, this will be derived as date of discharge – the date of admission + 1 – the days not-required.  - For infants who were still inpatient during the data collection period, this will be the date of the last daily collection form – the date of admission + 1 – the days not-required. |
| Number of days infant had NEWS-K CNMC completed during hospital stay | The sum of number of times ‘Yes’ was answered for “was the NEWS-K CNMC completed today” on daily data collection forms for an infant. |
| Percentage of days infant had NEWS-K CNMC completed | This will be calculated by dividing the number of days infant had NEWS-K CNMC completed during hospital stay by the number of days infant should have NEWS-K CNMC completed. |
| NEWS-K CNMC completed/not-completed/not-required on the 1^st^/2^nd^/…/7^th^ day in newborn unit | The number of infants who had NEWS-K CNMC completed/not-completed/not-required on the 1^st^/2^nd^/…/7^th^ of their hospital stay.  Percentages will be calculated using the number of infants in hospital on the day as denominator. |
| Number of days infants had observations on NEWS-K CNMC during morning/afternoon/night | The sum of number of times the recorded number of sets of vital signs is ≥ 1 during morning/afternoon/night on daily data collection form. |
| Percentage of days infants had NEWS-K CNMC completed during morning/afternoon/night | This will be calculated by dividing the number of days infants had observations on NEWS-K CNMC during morning/afternoon/night by the number of days infant should have NEWS-K CNMC completed during hospital stay. |
| Average number of (sets of) vital sign observations per infant per day taken on NEWS-K CNMC | Infant’s total number of (sets of) vital sign observations will be the sum of the number of (sets of) vital signs recorded on daily data collection form during hospital stay.  Average number of (sets of) vital sign observations per day will be calculated by dividing infant’s total number of (sets of) vital sign observations by the number of days infant should have NEWS-K CNMC completed. |
| Average number of (sets of) vital signs observations per infant per day taken during morning/afternoon/night | Infant’s total number of (sets of) vital sign observations during morning/afternoon/night will be the sum of the number of (sets of) vital signs recorded during morning/afternoon/night on daily data collection form during hospital stay.  Average number of (sets of) vital sign observations per day will be calculated by dividing infant’s total number of (sets of) vital sign observations during morning/afternoon/night by the number of days infant should have NEWS-K CNMC completed. |
| Total number of (sets of) vital sign requiring escalation in amber/red zone | The sum of the number of (sets of) vital sign recorded in amber/red zone on the daily data collection form. |
| Total number of observation requiring escalation in red/amber by other vital signs | The derived variable will be the sum of the number of *xx* observations recorded on infants’ escalation and outcome form in amber/red zone, where *xx* =:   - Pulse - Respiratory rate - O2 sats (on O2) - O2 sats (on air) - Blood sugar |
| Total number of temperature observations requiring escalation in high/low amber/red zone | Temperature requiring escalation in high/low amber/red zone will be defined according to the following criteria:  Low amber: 35.6 – 36.4  Low red: < 35.5  High red: > 37.5  The derived variable will be the sum of the number of temperature observations recorded on infants’ escalation and outcome form in amber/red zone |
| Clinical response as per local guideline documented | This will be derived from the details entered in the escalation and outcome form. |
| Ratio of number of staffs and infants during morning/afternoon/night according to level of care | This will be calculated by the number of infants in level 1/2/3 during morning/afternoon/night divided by the number of staffs during morning/afternoon/night. |

- 1. **Procedures for missing data**

All analyses will be for participants with data collected. There will be no imputation for missing data.

1. **DESCRIPTION OF PARTICIPANT CHARACTERISTICS**
   1. **Participant flow**

The flow of infants through the study will be summarised in a flow diagram which will show the numbers of infants admitted to the newborn unit, the number of infants born < 37 weeks gestation and/or weighing < 2500 grams, the number of infants born ≥ 37 weeks gestation and weighing ≥ 2500 grams, the number of infants not entered into the observational study, the number of infants with data collected in the observational study, and the number of infants with data collected and eligible (birth weight < 2500g and/or < 37 weeks gestation). In addition, the number of eligible infants with data collected will be presented by hospitals and overall.

- 1. **Baseline characteristics**

Infant characteristics at being admitted to the newborn units will be presented overall and for each hospital, which are described as follows:

- Infant sex
- Place at birth
- Infant birthweight (grams)
- Estimated gestation age at birth (weeks)
- Methods of gestation age estimation
- Mode of delivery
- Times between birth and admission to newborn unit (hours)
- Main reason for admission
- Other reasons for admission (not mutually exclusive)
  1. **Infant outcome**

Infant outcomes at the end of data collection period will be collected and presented for each hospital and overall, which will be described in the table below:

| **Infant outcome** | **Description** |
| --- | --- |
| Infant status summary | Infant vital status at the end of the data collection period (died, discharged, inpatient at the end of the data collection period). |
| Outcome for infants that died before discharge | Including time between birth and death, and cause of death. |
| Outcome for infants discharged home | Including length of hospital stay, weight at discharge, and diagnoses during hospital stay. |
| Outcome for infants still inpatient at the end of data collection period | The length of infant hospital stay from admission to the end of the data collection period. |

Continuous data will be summarised in terms of the mean, standard deviation, median, lower and upper quartiles, minimum, maximum, and number of observations. Categorical data will be summarised in terms of frequency counts and percentages.

1. **ANALYSIS OF FEASIBILITY OF NEWS-K**

Outcomes relating to the feasibility of the `NEWS-K CNMC’ form’ will be summarised descriptively with outcomes summarised overall and by hospital. Triggers and escalation outcomes will also be summarised by time of day and vital signs. Percentages will be presented with 95% confidence intervals. For the key feasibility outcomes as descried below:

- Percentage of days infants had NEWS-K CNMC completed during hospital stay
- Percentage of issues escalated to a more senior member of staff as per guidance on reverse of NEWS-K CNMC
- Percentage of days infants had observations on NEWS-K CNMC during morning/afternoon/night
- Percentage of issue escalated as per guidance on reverse of NEWS-K CNMC during morning/afternoon/night

The infant characteristics will be compared between the infants whom the NEWS-K CNMC was completed and the infants whom the NEWS-K CNMC was not completed. Infants who had CNMC completed at least 50% of the days that they should have CNMC completed during hospital stay will be classified as completed. The comparisons will be conducted for each hospital and overall.

Three indicators will be applied to assist to determine the feasibility of proceeding to a clinical trial, including the completion of the NEWS-K CNMC form, the escalation of care according to the NEWS-K CNMC, and the time of completion of the NEWS-K CNMC. The following criteria is proposed:

| **Indicator** | **RED** | **AMBER** | **GREEN** |
| --- | --- | --- | --- |
| 1. **NEWS-K CNMC form is completed** | **<60%** of eligible infants had vital signs recorded using the NEWS-K CNMC form on at least **50%** of  the days CNMC should have been completed | **>60%** - < 75% of eligible infants had vital signs recorded using the NEWS-K CNMC form on at least **50%** of the  days CNMC should have been completed | **>75%** of eligible infants had vital signs recorded using the NEWS-K CNMC form on at least **50%** of the  days CNMC should have been completed |
| 1. **Escalation of care, according to the NEWS-K CNMC form** | Care was escalated **<40%** of the time where care was required to be escalated to a more senior member of staff in accordance with the NEWS-K CNMC form | Care was escalated **>40% - <60%** of the time where care was required to be escalated to a more senior member of staff in accordance with the NEWS-K CNMC form | Care was escalated **>60%** of the time where care was required to be escalated to a more senior member of staff in accordance with the NEWS-K CNMC form |
| 1. **Time of completion of the NEWS-K CNMC form** | Completion of the NEWS-K CNMC form and subsequent escalation of care (as required) very dependent upon the time of day; defined as **>75%** difference between completion in morning, afternoon and night | Completion of the NEWS-K CNMC form and subsequent escalation of care (as required) dependent upon the time of day; defined as >50% - **<75%** difference between completion in morning, afternoon and night | Completion of the NEWS-K CNMC form and subsequent escalation of care (as required) is not dependent upon the time of day; defined as **<50%** difference between completion in morning, afternoon and night |

|  | * If one or more indicator is in the red zone, feasibility has not been demonstrated and it is not possible to proceed to a clinical trial | * If one or more indicator is in the amber zone, it may be possible to proceed to a clinical trial if significant modifications are implemented | * If all three indicators are in the green zone, it will be possible to proceed to a clinical trial. If less than all three indicators are green, some modifications may be required in order to proceed to a clinical trial. |
| --- | --- | --- | --- |

In addition, the number of staffs on shift and the number of infants in the newborn units will be collected according to level of care for each hospital. Definition of level of care is described as bellow:

| **Level of care** |  |
| --- | --- |
| 1. **Level 1** | **Babies on oxygen/continuous positive airway pressure (CPAP) or intravenous fluids, who are acutely ill and unstable and require the closest monitoring and a higher level of care.** |
| 1. **Level 2** | **Babies who have stabilised but may still be ill and receiving, for example, assisted feeding and intravenous drugs or who require close monitoring.** |
| 1. **Level 3** | **Babies who are stable requiring only monitoring or oral medication often after steeping down from level 1 and 2 care. These babies may require regular feeding but limited care in terms of nursing observations.** |

Data will be summarised descriptively for each hospital and overall as well as by time. Ratios between staffs and infants will also be calculated.

1. **ANALYSIS OF EFFECTIVENESS/EFFICACY**

Not applicable.

1. **HEALTHCARE PROFESSIONAL EXPERIENCE ANALYSIS**

An additional analysis will be conducted on healthcare professional’s experience of using the ‘NEWS-K CNMC’ to further investigate the feasibility of the NEWS-K CNMC. Health professionals who were involved in the management and delivery of the neonatal care services during the NEWS-K study data collection period will be included.

Data will be collected through the questionnaire sent online to the participants at the end of the data collection period.

The outcomes will be whether the healthcare professionals have completed the NEWS-K CNMC during the NEWS-K study, and their experience of using the NEWS-K CNMC for monitoring infants. The analysis methods for outcomes will be descriptive.

Baseline characteristics for the healthcare professionals will also be presented overall and each hospital; including role of the healthcare professionals, time of working in newborn care units, and whether they have used CNMC to monitor infants. Continuous data will be summarised in terms of the mean, standard deviation, median, lower and upper quartiles, minimum, maximum, and number of observations. Categorical data will be summarised in terms of frequency counts and percentages.

1. **FINAL REPORT TABLES AND FIGURES**

See separate dummy table document: 1933 NEWS-K Dummy Tables for final analysis final version 1.0 21012022 .docx

1. **REFERENCES**
